# Prevalence of Vaccine-Covered and Non-Covered HPV Genotypes Among Unvaccinated Women in Ankara: A Single-Center Study

**DOI:** 10.3390/vaccines13060640

**Published:** 2025-06-13

**Authors:** Ayfer Bakır, Mehmet Alican Sapmaz

**Affiliations:** 1 Department of Medical Microbiology, Ankara Etlik City Hospital, University of Health Sciences, Ankara 06170, Türkiye; 2 Department of Obstetrics and Gynecology, Ankara Etlik City Hospital, University of Health Sciences, Ankara 06170, Türkiye; dr.alicansapmaz@hotmail.com

**Keywords:** human papillomavirus, genotype, polymerase chain reaction, cervical smear, vaccine, prevalence

## Abstract

Background/Objectives: Understanding the regional distribution of human papillomavirus (HPV) genotypes is essential for guiding effective vaccination and screening strategies. This study aimed to assess the prevalence and distribution of HPV genotypes among unvaccinated women aged 30 years and older undergoing routine screening in Ankara. It also aimed to compare the frequencies of genotypes included and not included in current vaccines and to investigate their association with cervical smear cytology. Methods: This descriptive, cross-sectional, single-center study was conducted at Ankara Etlik City Hospital between 15 November 2024 and 15 February 2025. A total of 500 sexually active, unvaccinated women aged 30 years or older were enrolled. Cervical swab samples were analyzed for HPV DNA and genotypes using real-time PCR (28-type panel), and cytology results were retrospectively obtained from medical records. Results: HPV infection was detected in 18.2% of participants. Among HPV-positive women, 71.4% had single-type and 28.6% had multiple-type infections. The most common high-risk genotypes among HPV-positive individuals were HPV 16 (13.2%), HPV 18 (13.2%), and HPV 59 (13.2%). While 35.2% of HPV-positive cases included genotypes covered by the nonavalent vaccine, 64.8% involved at least one genotype not covered, mainly HPV 59, 44, and 51. HPV was detected in 17% of individuals with normal cytology, 19% of those with atypical squamous cells of undetermined significance (ASC-US), and 100% of cases with low-grade squamous intraepithelial lesion (LSIL) (*p* < 0.001). Conclusions: The findings emphasize the persistence of high-risk and non-vaccine-covered HPV types in the population, highlighting the need for updated vaccination policies and the development of broader-spectrum vaccines aligned with local genotype profiles.

## 1. Introduction

Human papillomavirus (HPV) is a member of the *Papillomaviridae* family, characterized by a double-stranded, circular DNA genome, and it exclusively infects humans [1].

To date, approximately 450 different HPV types have been identified, and 12 of them (HPV 16, 18, 31, 33, 35, 39, 45, 51, 52, 56, 58, and 59) are known to have high carcinogenic potential [2]. HPV 16 and HPV 18 are responsible for approximately 70% of cervical cancer cases and also play a significant role in anal, head and neck, and other genital cancers [3]

Globally, cervical cancer is the fourth most common cancer among women, with a particularly high prevalence in low- and middle-income countries [4]. According to 2022 GLOBOCAN data, approximately 604,000 new cases of cervical cancer are diagnosed globally each year, resulting in around 342,000 deaths [5].

HPV infection is transmitted through sexual contact. Although most infections are transient and cleared by the immune system within one to two years, approximately 1–3% may persist and gradually progress to cervical precancer or cancer over several years [6].

The most effective method for preventing HPV infection is vaccination with HPV vaccines containing L1 virus-like particles (VLPs), produced using recombinant DNA technology [7]. The distribution of HPV genotypes varies significantly across countries and regions, necessitating the adaptation of vaccination and screening strategies to local epidemiological characteristics [8].

The primary aim of HPV vaccines is to prevent invasive cervical cancer, which is achieved by providing protection against high-risk HPV types. As of December 2019, 124 countries worldwide had launched national HPV vaccination programs [9]. Three prophylactic HPV vaccines have been licensed globally: the quadrivalent, bivalent, and nonavalent vaccines, approved in 2006, 2007, and 2014, respectively [10]. The currently available vaccines include the bivalent vaccine (Cervarix^®^; targeting HPV 16 and 18), the quadrivalent vaccine (Gardasil^®^; targeting HPV 6, 11, 16, and 18), and the nonavalent vaccine (Gardasil 9^®^; targeting HPV 6, 11, 16, 18, 31, 33, 45, 52, and 58). The broader genotype coverage of the nonavalent vaccine has reduced the need for cross-protection [11]. It is estimated that the nonavalent HPV vaccine could prevent approximately 90% of cervical cancer cases globally [12].

The bivalent and quadrivalent HPV vaccines offer limited cross-protection against non-targeted oncogenic HPV types. To expand genotype coverage, nonavalent vaccines have been developed, and clinical trials for 11-valent vaccine candidates are currently ongoing [10,13,14]. Ten-year follow-up studies on the 9-valent HPV vaccine have demonstrated high levels of efficacy and safety [15]. Nevertheless, HPV vaccination coverage worldwide remains below the desired levels. Although the World Health Organization has set a target for 90% of girls to be fully vaccinated against HPV by 2030, global first-dose vaccination coverage declined from 25% to 15% between 2019 and 2021 [16]. In Türkiye, HPV screening is conducted through the national cervical cancer screening program, but HPV vaccination is not yet part of the routine immunization program.

This study aims to determine the prevalence and distribution of HPV genotypes among unvaccinated women aged over 30 in Ankara; to compare the frequency of genotypes covered and not covered by current vaccines; and to evaluate the association between HPV genotypes and abnormal cervical cytology findings.

## 2. Materials and Methods

### 2.1. Study Design

This study was conducted between 15 November 2024 and 15 February 2025 at the Departments of Obstetrics and Gynecology and Medical Microbiology of Etlik City Hospital in Ankara, Türkiye. The population included sexually active women aged 30 years and older who voluntarily participated after presenting for routine cervical cancer screening.

Designed as a descriptive cross-sectional survey, the study did not include a separate control group. However, differences in HPV status (positive vs. negative) among participants allowed for internal comparisons within the cohort, consistent with the study’s observational design.

Cervical swab samples were collected by a specialist physician using a flocked swab and transferred into a Biospeedy viral nucleic acid transport (VNAT) tube containing 3 mL of transport medium. The samples were subsequently transported to the molecular microbiology laboratory. Samples not processed for HPV PCR analysis within five days were stored at –20 °C to preserve nucleic acid integrity.

### 2.2. Inclusion and Exclusion Criteria

The study included women who presented to the Obstetrics and Gynecology Clinic for routine cervical cancer screening and met the following criteria: aged 30 years or older, sexually active, had not previously received HPV vaccination, agreed to provide cervical swab samples, signed an informed consent form, and voluntarily agreed to participate in the study. Women aged 30 years and older were included in accordance with the guidelines of the Turkish National Cervical Cancer Screening Program, which targets this age group for HPV testing.

Exclusion criteria were as follows: being under the age of 30; current pregnancy, postpartum, or lactation period; diagnosis of advanced gynecological disease or cervical cancer; a history of cervical cancer treatment; receiving immunosuppressive therapy; or undergoing active treatment for another malignancy.

### 2.3. Molecular Analysis

All cervical swab samples were transferred into VNAT™ transport tubes (Cat. No: NN-135) prior to analysis and stored at temperatures between +2 °C and +8 °C, in accordance with the manufacturer’s instructions. Since the samples stabilized in NAT tubes did not require nucleic acid extraction, they were directly subjected to quantitative polymerase chain reaction (qPCR) analysis.

For the detection and genotyping of HPV DNA in cervical samples, a commercially available HPV Genotyping qPCR Kit (Cat. No: BS-GHPV-1-25/BS-GHPV-1-100; Bioeksen R&D Technologies Inc., Istanbul, Türkiye) was used. This panel operates on the principle of real-time polymerase chain reaction and enables the qualitative detection of a total of 28 distinct HPV genotypes.

The HPV genotypes were categorized into three risk groups: high-risk, probable high-risk, and low-risk types. The classification of genotypes according to their oncogenic risk level, as well as their inclusion in currently available HPV vaccines (bivalent, quadrivalent, and nonavalent), is summarized in Table 1.

The analyses were performed using the Magnetic Induction Cycler (MIC) device manufactured by Bio Molecular Systems (Coomera, QLD, Australia), in accordance with the manufacturer’s protocol. The sensitivity and specificity of the kit for HPV genotype detection were 99.5% and 99.48%, respectively. Each run included a negative control (NTC), a positive control (PC), and an internal control (IC).

Samples that exhibited signal detection above the threshold and demonstrated a sigmoidal amplification curve were interpreted as positive. In contrast, samples with no amplification curve were considered negative. The validity of the results was assessed using automated analysis via the Sigmoida Software (V 8.6 REV.56) integrated into the device (Bioeksen R&D Technologies Inc., Istanbul, Türkiye), with manual interpretation performed when necessary to support result accuracy.

### 2.4. Cytological Evaluation

Cervical smear results were retrospectively obtained from the hospital’s electronic medical record system. Cytological findings were classified according to the Bethesda System as follows: negative for intraepithelial lesion or malignancy (NILM), atypical squamous cells of undetermined significance (ASC-US), low-grade squamous intraepithelial lesion (LSIL), high-grade squamous intraepithelial lesion (HSIL), and atypical glandular cells (AGC).

### 2.5. Statistical Analysis

All data were analyzed using IBM^®^ SPSS^®^ Statistics for Windows, Version 25.0 (IBM Corp., Armonk, NY, USA). The demographic characteristics of the participants and the distribution of HPV genotypes were summarized using descriptive statistical measures such as mean, median, and standard deviation. Differences between HPV-positive and HPV-negative groups were evaluated using appropriate statistical tests based on the type of variables. Categorical variables were analyzed using the Chi-square test, while continuous variables were assessed using the independent samples *t*-test for normally distributed data and the Mann–Whitney U-test for non-normally distributed data. A *p*-value < 0.05 was considered statistically significant.

## 3. Results

A total of 500 women aged 30 years and older were screened for HPV genotypes and cervical cytology. Among the participants, 83.4% (n = 417) were in the 30–49 age group, and all were married. The majority of participants (96.6%; n = 483) were of Turkish nationality.

### 3.1. HPV Prevalence and Most Frequently Detected Genotypes

**Figure 1:**
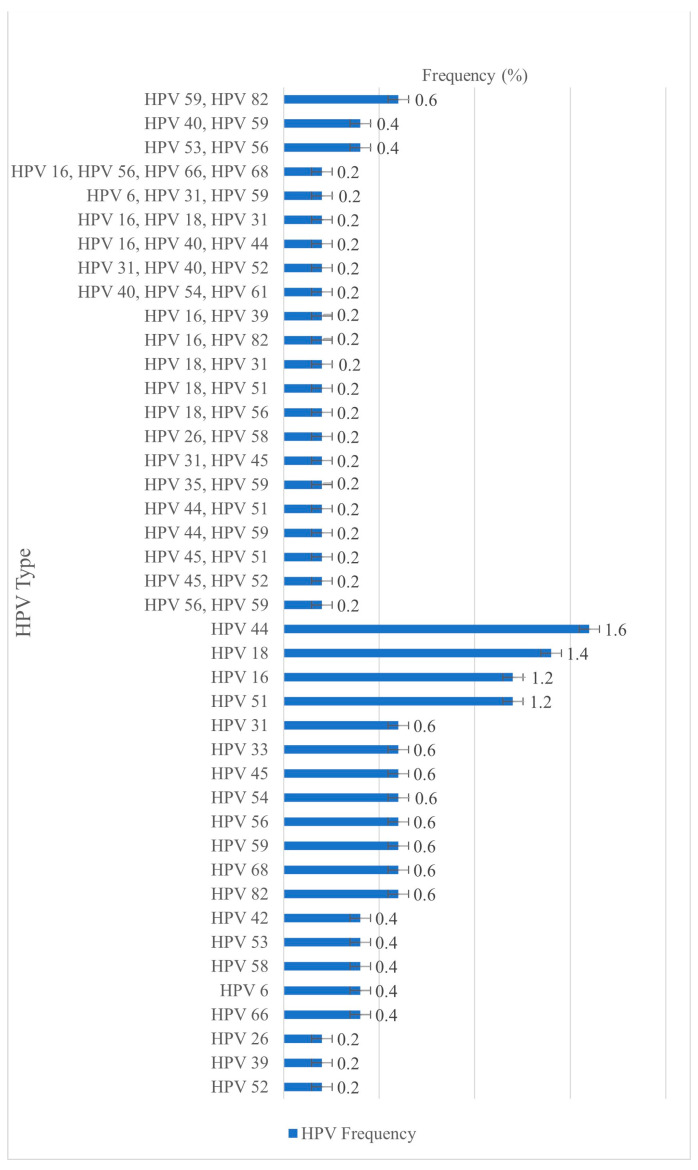
Distribution of detected HPV genotypes by infection type among all participants.

The overall prevalence of HPV infection was 18.2% (91/500; 95% CI: 14.9–21.5%). Among HPV-positive individuals, 71.4% (n = 65) were infected with a single HPV genotype, while 28.6% (n = 26) had multiple infections involving more than one genotype (Figure 1).

Among all participants (n = 500), high-risk (HR) HPV types were detected in 12.6% (n = 63), low-risk (LR) types in 4.8% (n = 24), and probable high-risk (PHR) types in 3.8% (n = 19). Among HPV-positive women (n = 91), HR genotypes accounted for 69.2%, LR genotypes for 26.4%, and PHR genotypes for 20.9%.

The most frequently detected high-risk HPV types were HPV 16 (2.6%), HPV 18 (2.6%), HPV 59 (2.4%), HPV 51 (2.0%), and HPV 31 (1.8%). In the LR group, the most common type was HPV 44 (2.2%), followed by HPV 40 (1.2%) and HPV 54 (1.0%). Among PHR types, the most frequently detected were HPV 82 (1.4%), HPV 53 (0.8%), and HPV 68 (0.8%) (Table 2).

The total number of HPV genotypes detected among the 91 HPV-positive cases was 126 due to the presence of multiple genotypes in some individuals. Of these, 68.3% (n = 86) were classified as high-risk (HR), 10.3% (n = 13) as probable high-risk (PHR), and 21.4% (n = 27) as low-risk (LR). HR types were found to be predominant both among all participants (n = 500) and within HPV-positive cases (n = 91).

**Figure 2:**
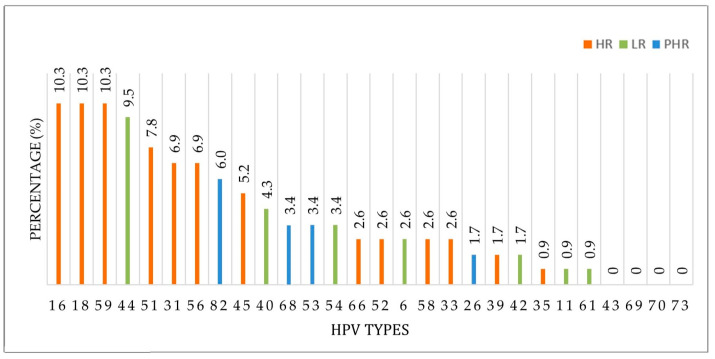
Distribution and risk classification of positive HPV genotypes detected among 126 HPV types. Note: Percentages were calculated based on 126 total HPV genotypes detected in 91 HPV-positive individuals.

The distribution, calculated based on the total of 126 identified HPV genotypes, is presented in Figure 2. Among the 126 identified HPV types, the most frequently detected HR genotypes were HPV 16 (10.3%), HPV 18 (10.3%), and HPV 59 (10.3%). The most commonly detected PHR type was HPV 44 (9.5%), while the most prevalent LR type was HPV 82 (6.0%).

### 3.2. Age-Based Distribution of HPV Infections

The prevalence of HPV positivity was evaluated across two age groups: 30–49 years and ≥50 years. HPV infection was detected in 20.4% (85/417) of women aged 30–49 and in 7.2% (6/83) of those aged ≥50, representing a statistically significant difference (*p* = 0.005).

Among women aged 30–49, the most frequently identified genotypes were HPV 59 (2.9%), HPV 18 (2.6%), HPV 44 (2.6%), and HPV 16 (1.4%). In contrast, among those aged ≥50 years, the most prevalent genotypes were HPV 16 (7.2%), HPV 18 (1.2%), and HPV 31 (1.2%) (Table 3).

Both single and multiple HPV infections were more commonly observed in the 30–49 age group. In this group, the prevalence of single-type infections was 14.1% (59/417) and multiple-type infections was 6.2% (26/417). Among women aged ≥50, the corresponding rates were 7.2% (6/83) for single-type and 1.2% (1/83) for multiple-type infections, also showing a statistically significant difference (*p* = 0.016).

### 3.3. Distribution of HPV Infections According to Vaccine Coverage

When classified according to currently available HPV vaccines, 14.3% (n = 13) of the 91 HPV-positive cases were fully covered by the bivalent vaccine, 17.6% (n = 16) by the quadrivalent vaccine, and 35.2% (n = 32) by the nonavalent vaccine (Table 2). In contrast, 64.8% (59/91) of the cases harbored at least one HPV genotype not included in any of the existing vaccines.

Among HPV-positive cases, the most frequently detected genotypes not covered by the nonavalent vaccine were HPV 59 (9.5%), HPV 44 (8.7%), and HPV 51 (7.1%) (Table 4).

### 3.4. Cytological Findings and Distribution of Vaccine-Covered and Non-Covered HPV Genotypes

Among participants with normal cytology, 17% (77/452) tested HPV-positive, compared to 19% (8/42) in the ASC-US group and 100% (6/6) in the LSIL group (*p* < 0.001).

Among the eight ASC-US cases, 87.5% (n = 7) had single-type HPV infections, and 12.5% (n = 1) had multiple-type infections. In contrast, among the six LSIL cases, 33.3% (n = 2) had single infections, while 66.7% (n = 4) were associated with multiple infections.

**Figure 3:**
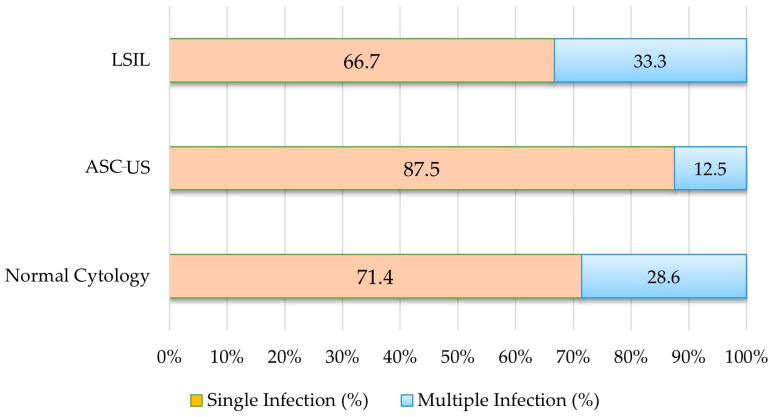
Distribution of single and multiple HPV infections according to cytological diagnosis. Note: Percentages were calculated based on the number of HPV-positive individuals in each cytological category (normal cytology: n = 77; ASC-US: n = 8; LSIL: n = 6).

The LSIL group had the highest proportion of multiple infections among all cytological subgroups (Figure 3).

Among the 77 HPV-positive individuals with normal cytology, 71.4% (n = 55) had single-type infections, while 28.6% (n = 22) had multiple-type infections. Although multiple HPV infections were more prevalent in the LSIL group, single-type infections were dominant among individuals with ASC-US and normal cytology (*p* = 0.066) (Figure 3).

In the normal cytology group, the most frequently detected genotypes were HPV 59 (10.4%), HPV 44 (9.4%), HPV 16 (9.4%), and HPV 18 (8.5%). In the ASC-US group, HPV 56 (22.2%) and HPV 18 (22.2%) were most commonly identified. Among women with LSIL, HPV 16 (18.2%) and HPV 45 (18.2%) were the predominant types detected (Table 5).

A considerable proportion of women with abnormal cytology were infected with HPV genotypes targeted by the nonavalent vaccine. In contrast, coverage by the quadrivalent and bivalent vaccines was notably lower. Among the six LSIL cases, four (66.7%) harbored HPV types included in the nonavalent vaccine. In the ASC-US group, 3 of 8 cases (37.5%), and in the normal cytology group, 25 of 77 cases (32.5%) were infected with vaccine-covered genotypes (Table 5).

## 4. Discussion

This study evaluated the prevalence of HPV genotypes and age-specific infection patterns among unvaccinated women over the age of 30 in Ankara. The overall HPV prevalence observed in our study was 18.2%, which falls within the moderate-to-high range when compared with global averages.

According to a global meta-analysis by Bruni et al., HPV prevalence among women with normal cytology varies significantly by region. Rates have been reported as low as 4.7% in North America and around 7.2% in Western Europe, while prevalence reaches approximately 21.1% across Africa and exceeds 24% in sub-Saharan regions. In Latin America and the Caribbean, prevalence typically ranges from 16% to over 20%, with some areas reporting rates close to 25% [17]. In Asia, studies have shown that HPV prevalence is heterogeneous, with higher rates observed in developing regions such as South and Southeast Asia, often exceeding 10%, and comparatively lower rates in economically advanced countries such as Japan and South Korea. This variation suggests that regional disparities in socioeconomic status and access to healthcare services may significantly influence HPV prevalence patterns [17,18]. According to multicenter and hospital-based studies conducted in Türkiye, HPV DNA positivity rates demonstrate substantial regional variability, ranging from 2.4% to 80% [19]. This wide variation is primarily due to the inclusion of heterogeneous patient populations, encompassing not only women attending routine cervical screening but also individuals referred for diagnostic, therapeutic, or oncological purposes. In contrast, our study was based on a homogeneous sample consisting exclusively of unvaccinated women who presented for routine screening. This group may represent a population with elevated clinical or behavioral risk. Additionally, the high analytical sensitivity of the HPV detection method used in our study could have contributed to increased positivity rates. The urban socioeconomic environment of Ankara, a densely populated metropolitan area, may also facilitate greater HPV transmission.

Previous studies have similarly demonstrated that regional socioeconomic disparities and access to healthcare are critical determinants of HPV prevalence [20].

In our study, the most frequently detected high-risk HPV genotypes were HPV 16, HPV 18, and HPV 59. Among probable high-risk types, HPV 44 was the most common, while HPV 82 was predominant among low-risk genotypes. Notably, genotypes such as HPV 59, HPV 44, and HPV 51, none of which are included in the nonavalent vaccine, were also found to circulate in the study population at measurable levels. Globally, the most prevalent high-risk HPV genotypes have been reported as HPV 16, HPV 52, HPV 58, HPV 18, HPV 33, HPV 31, HPV 35, and HPV 45 [21]. The high detection rate of HPV 16 in our study aligns with these global trends. However, the notable presence of HPV 59 and HPV 44, which are not included in the current nonavalent vaccine, highlights the importance of regional variation in genotype distribution. The study by Zhao and colleagues also emphasized significant intercontinental differences. For instance, HPV 52 and 58 were more prevalent in Asia, whereas HPV 31 and 18 were more commonly reported in Europe and North America. These findings underscore the need to consider regional genotype profiles when designing HPV vaccination and screening strategies. Public health interventions that are tailored to local epidemiological patterns may enhance vaccine effectiveness, optimize resource allocation, and improve the real-world impact of immunization programs.

Similarly, a large-scale study conducted in the Guangzhou region of China reported HPV 52, HPV 16, and HPV 58 as the most frequently detected types. The study also identified HPV 53, HPV 68, HPV 51, and HPV 59 as the most common high-risk genotypes not included in the nonavalent vaccine, while HPV 81 was the most prevalent among the low-risk types [22]. Studies by Zhou et al. and Zhao et al. consistently identified HPV 52, HPV 16, and HPV 58 as the most frequently detected genotypes. In both studies, several HPV genotypes not included in the nonavalent vaccine were also detected at notable frequencies. Among these, HPV 53 and HPV 66 emerged as the most consistently prevalent high-risk types across both datasets, while HPV 81 was identified as the most common low-risk genotype [23,24]. Another study from Henan Province in China similarly reported HPV 16, HPV 52, and HPV 58 among the most frequently encountered types, while also identifying significant rates of nonavalent vaccine-excluded genotypes such as HPV 53 and HPV 66 [25]. In a large-scale study conducted in Hungary, HPV 16, HPV 31, and HPV 58 were reported as the most frequently detected high-risk genotypes. HPV 66 and HPV 68, which are not included in the nonavalent vaccine, were also identified at notable frequencies [26]. Likewise, a study from Ghana reported that, in addition to vaccine-covered types such as HPV 16, HPV 18, HPV 52, and HPV 58, nonavalent-excluded types such as HPV 35 and HPV 66 were also found in significant proportions [8]. These findings highlight the geographical variation in HPV genotype distribution and reinforce the importance of incorporating local epidemiological data into vaccination and screening strategies.

In this study, 14.3% of the 91 HPV-positive cases were found to carry genotypes targeted by the bivalent vaccine, 17.6% by the quadrivalent vaccine, and 35.2% by the nonavalent vaccine. Among the HPV-positive cases, the most common genotypes not covered by the nonavalent vaccine were HPV 59, HPV 44, and HPV 51. These findings suggest that vaccination programs may be effective in reducing the burden of HPV infection. However, the substantial presence of non-vaccine-covered types highlights the need to update vaccination strategies in accordance with regional epidemiological data. According to a systematic review, the bivalent and quadrivalent vaccines offer a certain degree of cross-protection against types targeted by the nonavalent vaccine, such as HPV 31, HPV 33, and HPV 45. However, this protection is limited and has been associated with a waning immune response over time [10]. Furthermore, large cohort studies have demonstrated that HPV vaccination at an early age reduces the risk of cervical cancer by up to 88% [9]. Similarly, a study by Rideg et al. reported that the most frequently detected genotypes in high-grade cervical lesion samples were HPV 16, HPV 51, HPV 33, HPV 58, and HPV 31. The particularly high prevalence of HPV 51, which is excluded from current nonavalent vaccines, supports the need to broaden vaccine coverage. Although our study was based on screening smear samples, and the study by Rideg et al. was based on histopathologically confirmed lesions, both studies identified a significant presence of high-risk HPV types not covered by current vaccines. This underscores the importance of continuously updating vaccination strategies to reflect regional genotype distribution [27].

In this study, the HPV positivity rate was analyzed by age group, revealing a prevalence of 20.4% among women aged 30–49 and 7.2% in those aged ≥50 (*p* = 0.005), indicating a statistically significant decrease in HPV prevalence with advancing age. In contrast, a population-based study conducted in Xi’an, China, reported the highest HPV prevalence in women aged ≥60 years (13.9%), with slightly lower rates observed in younger groups [20]. A study from Germany reported that HPV prevalence declined with age, with rates of 13.6% in the 30–39 age group, 10.3% in the 40–49 age group, 9.9% in the 50–59 age group, and 6.9% in women aged 60 years and older [28].

In this study, 71.4% of HPV DNA-positive samples involved single infections, while 28.6% were multiple infections. This pattern aligns with findings from both China and Germany, where single-type HPV infections were more common than multiple infections [20,28]. These consistent findings suggest that both the overall prevalence of HPV infection and the rate of multiple infections tend to decline with age, possibly reflecting improved immune control in older individuals. Collectively, this pattern highlights the importance of tailoring HPV screening strategies based on age and infection profile.

Continuous monitoring of HPV genotype distribution is essential for assessing the effectiveness of vaccination programs and for guiding the development of future vaccines. In our study, the notable presence of genotypes not covered by current vaccines emphasizes the need to update both screening and immunization policies in response to evolving epidemiological trends. However, the formulation and success of such updated immunization strategies critically depend on the accessibility and public adoption of HPV vaccination programs. In Türkiye, HPV vaccination is not currently included in the national immunization program and is therefore not offered free of charge. Individuals who wish to receive the vaccine must access it through private healthcare providers, usually upon recommendation by family physicians or gynecologists. Cost and limited access remain major barriers to widespread vaccination. According to a recent study, HPV vaccination coverage among women in Türkiye is estimated to be as low as 3.6%, underscoring the urgent need for more accessible, publicly funded vaccination strategies and increased awareness efforts [29].

The strengths of this study include the simultaneous evaluation of HPV DNA and cytological results, along with comprehensive genotyping in an unvaccinated population. However, its cross-sectional design limits conclusions about the natural history of infection, and the use of a screening-based sample may reduce the generalizability of the results. Additionally, the limited sample size and the fact that the study was conducted in a single tertiary hospital in Ankara further restrict the generalizability of the findings to the overall population of Türkiye. Since the primary aim of this study was to determine the distribution of HPV genotypes covered and not covered by vaccines, data on individual risk factors, such as sexual behavior, number of partners, or sexually transmitted disease (STD) history, were not collected. This is one of the limitations of the study.

## 5. Conclusions

Overall, this study provides valuable regional data on HPV genotype prevalence, patterns of multiple infections, and vaccine coverage. An HPV prevalence of 18.2% was found among unvaccinated women aged 30 years and older in Ankara, with high-risk genotypes continuing to circulate in the population. The detection of oncogenic types such as HPV 16, HPV 18, and HPV 59 supports the need to reinforce cervical cancer prevention efforts. In addition, the substantial presence of genotypes not covered by existing vaccines, particularly HPV 59, HPV 44, and HPV 51, underlines the importance of expanding vaccine formulations and advancing the development of new vaccines adapted to local genotype profiles.

## Figures and Tables

**Table 1:** Genotype coverage of the HPV genotyping qPCR panel used in this study and comparison with commercial HPV vaccines.

HPV Type	Risk Category	Bivalent	Quadrivalent	Nonavalent
HPV 6	Low risk		√	√
HPV 11	Low risk		√	√
HPV 16	High risk	√	√	√
HPV 18	High risk	√	√	√
HPV 26	Probable high risk			
HPV 31	High risk			√
HPV 33	High risk			√
HPV 35	High risk			
HPV 39	High risk			
HPV 40	Low risk			
HPV 42	Low risk			
HPV 43	Low risk			
HPV 44	Low risk			
HPV 45	High risk			√
HPV 51	High risk			
HPV 52	High risk			√
HPV 53	Probable high risk			
HPV 54	Low risk			
HPV 56	High risk			
HPV 58	High risk			√
HPV 59	High risk			
HPV 61	Low risk			
HPV 66	Probable high risk			
HPV 68	Probable high risk			
HPV 69	Probable high risk			
HPV 70	Low risk			
HPV 73	Probable high risk			
HPV 82	Probable high risk			

Note: HPV risk classifications are based on the International Agency for Research on Cancer (IARC) Monographs.

**Table 2:** HPV type combinations and infection types: comparison between total participants (N = 500) and HPV-positive women (n = 91).

Parameter	All Participants (N = 500)		HPV-Positive Cases (n = 91)	
	N (%)	95 Cl	n (%)	95 Cl
HPV positive	91 (18.2)	14.9–21.5		
Single infection	65 (13.0)	10.2–16.0	65 (71.4)	61.5–80.2
Multiple infection	26 (5.2)	3.4–7.2	26 (28.6)	19.8–38.5
Low-risk HPV genotype	24 (4.8)	3.2–7.0	24 (26.4)	18.4–36.3
Probable high-risk HPV genotype	19 (3.8)	2.4 –5.9	19 (20.9)	13.8–30.3
High-risk HPV genotype	63 (12.6)	10.0–15.8	63 (69.2)	59.1–77.8
Low-risk HPV genotype				
HPV 6	3 (0.6)	0.2–1.7	3 (3.3)	1.1–9.2
HPV 11	1 (0.2)	0.0–1.1	1(1.1)	0.2–6.0
HPV 40	5 (1.0)	0.4-2.3	5 (5.5)	2.4–14.2
HPV 42	2 (0.4)	0.1–1.4	2 (2.2)	0.6–7.7
HPV 43	0 (0.0)	0.0–0.8	0 (0.0)	0.0–4.1
HPV 44	11(2.2)	1.2–3.9	1(1.1)	0.2–6.0
HPV 54	4 (0.8)	0.3–2.0	4 (4.4)	1.7–10.8
HPV 61	1 (0.2)	0.0–1.1	1(1.1)	0.2–6.0
HPV 70	0 (0.0)	0.0–0.8	0 (0.0)	0.0–4.1
Probable high-risk HPV genotype				
HPV 26	2 (0.4)	0.1–1.4	2 (2.2)	0.6–7.7
HPV 53	4 (0.8)	0.3–2.0	4 (4.4)	1.7–10.8
HPV 66	3 (0.6)	0.2–1.7	3 (3.3)	1.1–9.2
HPV 68	4 (0.8)	0.3–2.0	4 (4.4)	1.7–10.8
HPV 69	0 (0.0)	0.0–0.8	0 (0.0)	0.0–4.1
HPV 73	0 (0.0)	0.0–0.8	0 (0.0)	0.0–4.1
HPV 82	7 (1.4)	0.7–2.9	7 (7.7)	3.8–15.0
High-risk HPV genotype				
HPV 16	12 (2.4)	1.4–4.1	12 (13.2)	7.7–21.6
HPV 18	12 (2.4)	1.4–4.1	12 (13.2)	7.7–21.6
HPV 31	8 (1.6)	0.8–3.1	8 (8.8)	4.5–16.4
HPV 33	3 (0.6)	0.2–1.7	3 (3.3)	1.1–9.2
HPV 35	1 (0.2)	0.0–1.1	1(1.1)	0.2–6.0
HPV 39	2 (0.4)	0.1–1.4	2 (2.2)	0.6–7.7
HPV 45	6 (1.2)	0.6–2.6	6 (6.6)	3.1–13.6
HPV 51	9 (1.8)	0.9–3.4	9 (9.9)	5.3–17.7
HPV 52	3 (0.6)	0.2–1.7	3 (3.3)	1.1–9.2
HPV 56	8 (1.6)	0.8–3.1	8 (8.8)	4.5–16.4
HPV 58	3 (0.6)	0.2–1.7	3 (3.3)	1.1–9.2
HPV 59	12 (2.4)	1.4–4.1	12 (13.2)	7.7–21.6
Vaccine genotypes				
*Bivalent*	13 (2.6)	1.5–4.4	13 (14.3)	8.5–22.9
*Quadrivalent*	16 (3.2)	2.0–5.1	16 (17.6)	11.1–26.7
*Nonavalent*	32 (6.4)	4.6–8.9	32 (35.2)	26.1–45.4

Abbreviations: N = total number of participants; n = number of HPV-positive cases; % = percentage; CI = 95% confidence interval; HPV = human papillomavirus. Prevalence values among all participants were calculated based on N = 500, while those among HPV-positive individuals were calculated based on n = 91. Since multiple HPV genotypes can be detected in a single individual, percentages in some categories may exceed 100%.

**Table 3:** Age-specific distribution of HPV types.

HPV Types	Age Groups (Years)						
	30–49 (N = 417)			≥50 (N = 83)			*p* Value
	n	%	95% CI	n	%	95% CI	
HPV 16	6	1.4	0.3–2.6	6	7.2	1.7–12.8	0.007
HPV 18	11	2.6	1.1–4.2	1	1.2	0.0–3.6	0.700
HPV 59	12	2.9	1.3–4.5	0	0.0	0.0–0.0	0.231
HPV 44	11	2.6	1.1–4.2	0	0.0	0.0–0.0	0.225
HPV 51	9	2.2	0.8–3.6	0	0.0	0.0–0.0	0.367
HPV 31	7	1.7	0.4–2.9	1	1.2	0.0–3.6	1.00
HPV 56	8	1.9	0.6–3.2	0	0.0	0.0–0.0	0.363
HPV 82	7	1.7	0.4–2.9	0	0.0	0.0–0.0	0.607
HPV 45	6	1.4	0.3–2.6	0	0.0	0.0–0.0	0.596
HPV 40	5	1.2	0.2–2.2	0	0.0	0.0–0.0	0.596
HPV 68	4	1.0	0.0–1.9	0	0.0	0.0–0.0	1.00
HPV 53	4	1.0	0.0–1.9	0	0.0	0.0–0.0	1.00
HPV 54	4	1.0	0.0–1.9	0	0.0	0.0–0.0	1.00
HPV 66	3	0.7	0.0–1.5	0	0.0	0.0–0.0	1.00
HPV 52	3	0.7	0.0–1.5	0	0.0	0.0–0.0	1.00
HPV 6	3	0.7	0.0–1.5	0	0.0	0.0–0.0	1.00
HPV 58	3	0.7	0.0–1.5	0	0.0	0.0–0.0	1.00
HPV 33	3	0.7	0.0–1.5	0	0.0	0.0–0.0	1.00
HPV 26	2	0.5	0.0–1.1	0	0.0	0.0–0.0	1.00
HPV 39	2	0.5	0.0–1.1	0	0.0	0.0–0.0	1.00
HPV 42	2	0.5	0.0–1.1	0	0.0	0.0–0.0	1.00
HPV 35	1	0.2	0.0–0.7	0	0.0	0.0–0.0	1.00
HPV 11	1	0.2	0.0–0.7	0	0.0	0.0–0.0	1.00
HPV 61	1	0.2	0.0–0.7	0	0.0	0.0–0.0	1.00
HPV 43	0	0.0	0.0–0.0	0	0.0	0.0–0.0	-
HPV 69	0	0.0	0.0–0.0	0	0.0	0.0–0.0	-
HPV 70	0	0.0	0.0–0.0	0	0.0	0.0–0.0	-
HPV 73	0	0.0	0.0–0.0	0	0.0	0.0–0.0	-

Note: N refers to the total number of women in each age group (30–49 years: N = 417; ≥50 years: N = 83). n refers to the number of individuals in whom each specific HPV genotype was detected. Percentages were calculated based on the total number of women in each respective age group.

**Table 4:** Most common HPV types not covered by vaccines.

HPV Types	HPV-Positive Case (n = 91)	
	n	%
HPV 59	12	9.5
HPV 44	11	8.7
HPV 51	9	7.1
HPV 56	8	6.3
HPV 82	7	5.6
HPV 40	5	4.0
HPV 68	4	3.2
HPV 53	4	3.2
HPV 54	4	3.2
HPV 66	3	2.4
HPV 39	2	1.6
HPV 26	2	1.6
HPV 42	2	1.6
HPV 35	1	0.8

Note: n indicates the number of individuals harboring the respective HPV genotype among the 91 HPV-positive cases. Only genotypes not covered by the nonavalent vaccine are included in this table.

**Table 5:** HPV genotype profile and vaccine coverage in HPV-infected women stratified by cytology.

HPV Type	Normal (n = 106)		ASC-US (n = 9)		LSIL (n = 11)	
	n	%	n	%	n	%
Vaccine type						
HPV 6	3	2.8	0	0.0	0	0.0
HPV 11	1	0.9	0	0.0	0	0.0
HPV 16	10	9.4	0	0.0	2	18.2
HPV 18	9	8.5	2	22.2	1	9.1
HPV 31	7	6.6	0	0.0	1	9.1
HPV 33	3	2.8	0	0.0	0	0.0
HPV 45	3	2.8	1	11.1	2	18.2
HPV 52	2	1.9	0	0.0	1	9.1
HPV 58	2	1.9	1	11.1	0	0.0
Non-vaccine type						
HPV 26	1	0.9	1	11.1	0	0.0
HPV 35	0	0.0	0	0.0	1	9.1
HPV 39	2	1.9	0	0.0	0	0.0
HPV 40	5	4.7	0	0.0	0	0.0
HPV 42	2	1.9	0	0.0	0	0.0
HPV 43	0	0.0	0	0.0	0	0.0
HPV 44	10	9.4	0	0.0	1	9.1
HPV 51	8	7.5	0	0.0	1	9.1
HPV 53	4	3.8	0	0.0	0	0.0
HPV 54	3	2.8	1	11.1	0	0.0
HPV 56	6	5.7	2	22.2	0	0.0
HPV 59	11	10.4	0	0.0	1	9.1
HPV 61	1	0.9	0	0.0	0	0.0
HPV 66	3	2.8	0	0.0	0	0.0
HPV 68	3	2.8	1	11.1	0	0.0
HPV 69	0	0.0	0	0.0	0	0.0
HPV 70	0	0.0	0	0.0	0	0.0
HPV 73	0	0.0	0	0.0	0	0.0
HPV 82	7	6.6	0	0.0	0	0.0
Vaccine genotype coverage						
Bivalent	10	13.0	2	25.0	1	17.0
Quadrivalent	12	16.0	3	38.0	1	17.0
Nonavalent	25	33.0	3	38.0	4	67.0

Note: Of the 126 HPV genotypes detected in total, 106 were identified in women with normal cytology, 9 in those with ASC-US, and 11 in those with LSIL. “n” values in the table represent the number of detections per genotype, not unique individuals.

## Data Availability

The data are not publicly available due to ethical and privacy restrictions.

## Abbreviations

The following abbreviations are used in this manuscript:

HPV	human papillomavirus
PCR	polymerase chain reaction
ASC-US	atypical squamous cells of undetermined significance
LSIL	low-grade squamous intraepithelial lesion
AGC	atypical glandular cells
HSIL	high-grade squamous intraepithelial lesion
HR	high-risk
LR	low-risk
PHR	probable high-risk
VLP	virus-like particle
